# Influence of Trail Running Footwear Foam on Running Economy and Perceptual Metrics

**DOI:** 10.1002/ejsc.70059

**Published:** 2025-09-27

**Authors:** Mélissa Muzeau, Andrew Flood, Nicholas Tam, Benoit Abel, Philo Saunders, Walter Staiano, Ben Rattray

**Affiliations:** ^1^ UCRISE University of Canberra Canberra Australia; ^2^ Discipline of Psychology University of Canberra Canberra Australia; ^3^ On AG Zurich Switzerland; ^4^ Athletics Australia Canberra Australia; ^5^ Department of Physical Education and Sport University of Valencia Valencia Spain; ^6^ Department of Psychology Biological and Cognitive Psychology University of Southern Denmark Odense Denmark

**Keywords:** efficiency, physiology, psychology, technology, testing

## Abstract

Advanced footwear technologies (AFT) improve road running performance. AFT have been incorporated into trail running footwear despite little evidence of their benefits in this context. In this study, we compared the effect of traditional (TRADI‐f) and AFT foam (AFT‐f) on running economy and perceptual measures across different gradients. Fourteen well‐trained athletes completed assessments on a treadmill at gradients of FLAT (0% gradient, 14 km.h^−1^), UP (+10%, 8 km.h^−1^) and DOWN (−10%, 14 km.h^−1^). Two shoes were randomly allocated in a counterbalanced order. The shoes were matched in construction but differed in midsole foam performance, where the AFT‐f shoe included a more compliant and resilient foam than the TRADI‐f shoe. Oxygen consumption and heart rate were collected for 6 min, twice with each shoe at each gradient, alongside perceived effort and affective measures. Across the three gradients, oxygen consumption was 1.2% lower (*p* = 0.008) when participants were wearing the AFT foam compared to the TRADI foam. The effect of the AFT‐f shoes on oxygen consumption appeared to be more pronounced in the FLAT (+2.1%) and UP (+1.0%) conditions compared to DOWN (+0.2%). This interaction effect was, however, not statistically significant (*p* = 0.050). RPE was lower (*p* = 0.008) and affective valence more positive (*p* = 0.027) in AFT‐f compared to TRADI‐f. No differences in arousal were reported between TRADI‐f and AFT‐f (*p* = 0.728). The findings of this study suggest that an AFT foam in trail running shoes can improve running economy, reduce perceived effort and increase pleasure while running in a trained athlete population.

## Highlights

- AFT foam improves running economy by ∼1%, with a tendency for greater benefits on level compared to uphill running.
- Specific attention should be given to protocol design to properly assess potential footwear‐related changes in running economy while running downhill.
- Affective responses should be more systematically integrated into product evaluation, as physiology alone does not capture the full spectrum of performance.

## 1 Introduction

Over the past decade, there has been a growing drive for innovation in road running, aimed at improving athletes' performance. More specifically, the hunt for marginal gains has led to advanced footwear technologies (AFT), characterised by midsoles that include a stiff curved plate and a lightweight, compliant and resilient foam (Frederick 2022; Burns and Joubert 2024). The specific combination of an increased longitudinal bending stiffness, improved energy return and rocker geometry has been shown to improve road running performance, with improvements of over 2% reported (Hébert‐Losier and Pamment 2022). These changes in performance can be considered alongside reported changes in running economy, with AFT enhancing running economy up to 4% (Hoogkamer et al. 2017; Hunter et al. 2019; Langley and Langley 2023). Running economy is a multifactorial concept encompassing the sum of metabolic, cardiorespiratory, neuromuscular and biomechanical factors impacting energy demand during steady‐state running (Barnes and Kilding 2015; Saunders et al. 2004). It has a strong predictive role in endurance performance and is typically reflected in a lower oxygen consumption and heart rate at a given steady‐state speed (Joyner and Coyle 2007).

Although evidence for the effects of AFT on running economy and performance has been reported in road running settings, the transferability to trail running remains unclear. Trail running involves long‐distance running across a succession of flat, uphill and downhill off‐road sections. Because of the unique demands of trail running, specific footwear has emerged. The unique demands of trail running also suggest that findings of the benefits of AFT in road running may not generalise to trail running. Preliminary data support this suggestion, indicating that the integration of a stiff plate in the midsole is beneficial in level running (Day and Hahn 2019; Chollet et al. 2022) but detrimental for uphill running (Jaboulay and Giandolini 2025). Further, AFT foam, thought to improve road running through improvement in running economy by a maximised return of mechanical energy (Worobets et al. 2014), is yet to be empirically tested in trail running conditions.

The reliance on changes in running economy as an indicator of the effects of AFT also limits the conclusions that can be drawn for trail running performance. Although running economy is one of the performance factors most likely impacted by footwear, the classical performance determinant model of long‐distance running (> 5 km) has been challenged in trail running. Composed of VO_2_ max, the percentage of VO_2_ max at threshold and running economy, the traditional performance determinant model predicts only 48% of performance in short‐distance trail running races (Scheer et al. 2018) compared to 97% in road running (McLaughlin et al. 2010). The predictive value of running economy for trail running performance remains inconclusive, as some studies suggest a predictive role (Scheer et al. 2018), whereas others do not (Balducci et al. 2017; Coates et al. 2021). As a result, the impact of footwear design for trail running footwear on other elements is worth considering for their predictive role of performance.

Millet (2011), in his model of ultradistance performance, highlighted the importance of psychological factors, including their interaction with equipment. Among these factors, the perception of effort plays a central role, especially in pacing and effort regulation (Tucker 2009; Millet 2011; Pageaux 2014; Renfree et al. 2012). The link between perceived effort and performance is well established: Lower perceived effort is generally associated with better performance (Marcora et al. 2009). A few studies were able to reduce perceived effort by manipulating affective state (Carmo et al. 2020; Blanchfield et al. 2014), also described as the most consciously accessible feeling one's experience. Anecdotally, AFT have been suggested to have a large impact on the athlete's feeling when running. However, research to date has focused primarily on footwear‐related metrics such as comfort (Mills et al. 2010; Matthias et al. 2021; Bishop et al. 2020; Hébert‐Losier et al. 2023), with limited emphasis placed on perceptual responses, including those related to effort and performance.

Given the recent advancements in footwear for trail running performance, the primary aim was to investigate the impact of trail running footwear foam type on running economy. Secondary aims sought to assess the impact of trail running footwear foam type on performance‐related perceptual measures of perceived effort and affect.

## 2 Methods

### 2.1 Study Design

The study used a randomised and counterbalanced repeated‐measures design with data collected through a single testing session. After expressing interest, participants received a link to sign up for the study, where inclusion/exclusion criteria were checked (see Participants section). Once admitted into the study, participants were sent requirements to follow 48 h prior to the test. They were asked to refrain from any strenuous physical activity, as well as alcohol and coffee consumption in the 24 h preceding the session, and to come properly rested and hydrated with the last meal ingested a minimum of 2 hours before. The aim of the study was explained to the participants as a comparison between two prototype shoes developed for the upcoming season, with no information provided regarding the differences between the two shoes. This study was approved by the Human Research Ethics Committee from the University of Canberra (No. 202413976). All procedures were conducted in accordance with the Declaration of Helsinki.

### 2.2 Participants

Eighteen trained to highly trained athletes, classified as Tiers 2 and 3 according to McKay et al. (2021) classification, volunteered to participate in this study. This sample size was chosen to replicate the study design of Hoogkamer et al. (2016). All participants met the inclusion criteria: > 18 years old, accustomed to uphill and downhill running, training > 30 km per week, reported being comfortable running at 16 km.h^−1^ and wearing EU shoe size 44. None reported musculoskeletal injuries impacting running performance. Four athletes were excluded after preliminary testing based on their physiological responses to a standardised speed (see the Experimental Procedure section). This resulted in a final sample size of 14 participants (age = 29.4 ± 7.3 years; height = 177.0 ± 3.7 cm; body mass = 68.5 ± 4.9 kg; weekly running distance = 78.9 ± 25.3 km).

### 2.3 Experimental Procedure

**FIGURE 1:**
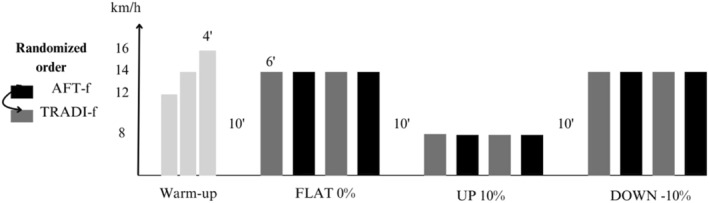
Experimental protocol.

Upon arriving at the laboratory, participants were requested to complete the compulsory part of the adult pre‐exercise screening system (Exercise and Sports Science Australia 2018) and the informed consent form. Participants initially completed preliminary testing on the treadmill while wearing their own shoes. Preliminary testing was used to assess eligibility for the study, a warm‐up and a familiarisation to the treadmill (h/p/cosmos gaitway 3D, Germany). To do so, participants ran for 4 minutes at speeds of 12 km.h^−1^, 14 km.h^−1^ and 16 km.h^−1 ‐^ with 30 s rest in between each 4‐min stage, where finger‐prick lactate was sampled (Lactate Scout, SensLab GmbH, Germany). Based on the team’s experience with athlete profiling, athletes were excluded from the study if an increase of +1.5 mmol.L^−1^ in blood lactate from 12 to 14 km.h^−1^ was observed, showing a clear change in lactate kinetics in order to account for intraindividual differences in absolute lactate values (*n* = 4). At speeds above the threshold, the slow component of VO_2_ implied that steady state was unlikely to be achieved during subsequent measurements, impacting the validity of the running economy measurement (Colosio et al. 2020). After this preliminary assessment, anthropometric measures were taken. Participants then put on the shoes that had been allocated to them for the first running economy assessment. The ordering in which each shoe was worn followed one of two sequences: A‐B‐A‐B or B‐A‐B‐A. The use of a shoe replicate in our study design followed the recommendations of Barrons et al. (2024). Participants then completed the running economy protocol. This protocol involved running for six minutes while oxygen consumption and heart rate were collected continuously. 30 s before the end of each trial, participants were asked to rate in the following order, while running, their perception of effort (6–20 Borg RPE; Borg 1998), affective valence (Feeling Scale; Hardy and Rejeski 1989) and arousal (Felt Arousal Scale; Svebak and Murgatroyd 1985). For each gradient (FLAT, UP, DOWN), participants completed the running economy protocol four times, changing shoes in between measurements, with a 3‐minute break allocated. A 10‐min break was provided between the first and the second gradient condition (FLAT to UP), as well as between the second and the third (UP to DOWN). Participants ran at 14 km.h^−1^ (0% incline) in the FLAT condition. The speed was selected based on the typical performance of elite long‐distance trailers. Those measurements were then replicated in UP at 8 km.h^−1^ (+10% incline) for its better performance prediction (Ehrström et al. 2017). The DOWN condition was assessed at 14 km.h^−1^ (−10% incline), as it was deemed the fastest speed ensuring participant safety while running on the treadmill (Figure 1).

### 2.4 Footwear and Mechanical Testing Protocol

**FIGURE 2:**
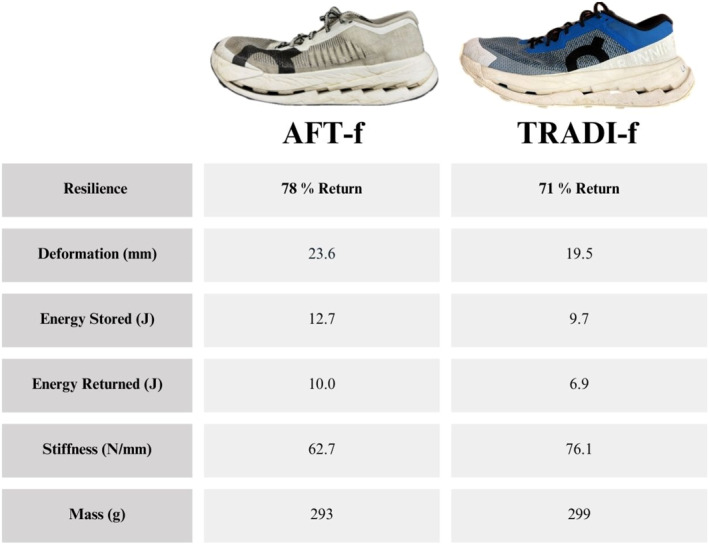
Mechanical properties of the footwear midsoles.

**FIGURE 3:**
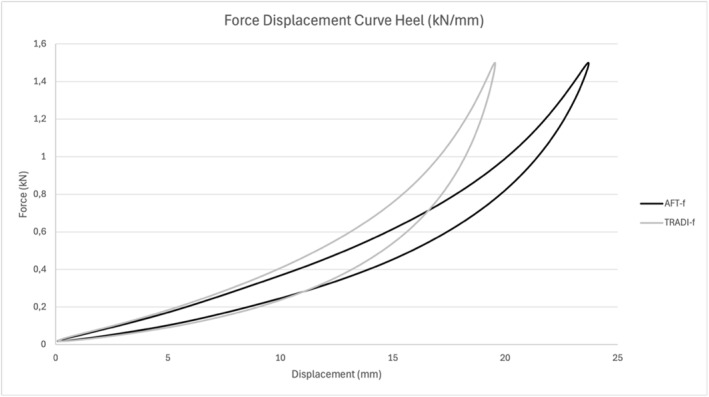
Mean force‐displacement curve of the midsoles assessed through servohydraulic testing (*n* = 2).

The two models of trail running footwear were similar in geometry with identical drop height and the inclusion of an AFT‐based plate. The difference in weight was considered negligible (Figure 2). The primary difference between the two shoes, apart from colour, was the midsole foam properties (Figure 2). The first shoe was considered a traditional foam (TRADI‐f) featuring stiffer foams, whereas the second shoe (AFT‐f) incorporated a highly resilient and compliant foam, characteristic of an AFT footwear (Figure 3). The mechanical properties of the midsoles were assessed using a servohydraulic testing device (LTM3, Zwick GmbH & Co. KG; Ulm, Germany). Each shoe condition was subjected to 60 consecutive sinusoidal loading‐unloading cycles with a 50‐mm diameter spherical pestle at a frequency of 2 Hz, with a maximum compressive load of 1500 N applied during each cycle. These test parameters were chosen to replicate typical vertical ground reaction forces experienced during running. Force and deformation data were recorded continuously at a sampling frequency of 1000 Hz through the testing procedure. The deformation (compliance) and hysteresis (resilience) of the footwear midsoles were quantified based on the data from the last 10 cycles.

### 2.5 Measurements

#### 2.5.1 Running Economy: Data Collection and Processing

Expired gases and heart rate were collected continuously during the running economy procedure. Expired gases were collected and analysed through a face mask attached to a portable metabolic cart (K5, COSMED, Italy). Heart rate was collected through a chest sensor (H10, Polar, Finland) synchronised with the metabolic cart. VO_2_, VCO_2_ and heart rate data were sampled over an averaged 10‐s binned time period. Ventilation rate and gas concentration were calibrated prior to each participant testing session. Additional gas concentration (O_2_ and CO_2_) was calibrated before each gradient condition (FLAT, UP, DOWN) according to the manufacturer's recommendations. Mixing chamber mode was selected given the steady‐state nature of the measurements. This allowed a more comfortable setting for the participants, more stable measurement and a lower source of errors (Ward 2018; Beijst et al. 2012; Perez‐Suarez et al. 2018). Running economy was reported as oxygen consumption (mL^−1^. kg^−1^. min^−1^). Heart rate was reported in beats per minute (bpm) as an additional physiological metric related to running economy. Energetic cost (W.kg^−1^) was also calculated through the energy equivalent table from Péronnet and Massicotte (1991) using respiratory exchange ratio (RER). Although not analysed, descriptive values for energetic cost are reported in Table 1 to allow comparison with existing research in the field (Hoogkamer et al. 2017; Chollet et al. 2022).

**TABLE 1:** Descriptive and statistical results with mean ± SD for each condition.

	FLAT		UP		DOWN		Shoe effect (*p* = )	df
	TRADI‐f	AFT‐f	TRADI‐f	AFT‐f	TRADI‐f	AFT‐f		
VO_2_ (mL.kg^−1^.min^−1^)	51.10 ± 3.16	50.04 ± 3.41	48.07 ± 2.00	47.57 ± 2.08	37.05 ± 2.78	36.99 ± 2.47	0.008^a^	77
Energetic cost (W.kg^−1^)	18.05 ± 1.15	17.66 ± 1.20	16.92 ± 0.68	16.73 ± 0.73	12.76 ± 0.98	12.73 ± 0.87		
Heart rate (bpm)	156 ± 13	155 ± 13	151 ± 14	149 ± 14	130 ± 12	128 ± 12	< 0.001^a^	61
RPE (6–20)	11.9 ± 1.9	11.5 ± 1.7	11.5 ± 2.0	11.3 ± 2.0	9.6 ± 1.2	9.6 ± 1.4	0.008^a^	79
Affective valence (−5 to 5)	2.5 ± 1.4	2.7 ± 1.3	2.4 ± 1.4	2.7 ± 1.2	2.6 ± 1.6	3.0 ± 1.5	0.027^a^	79
Arousal (1–6)	2.8 ± 0.9	2.7 ± 0.9	2.8 ± 1.2	2.7 ± 1.1	3.3 ± 1.3	3.3 ± 1.3	0.738	79

Abbreviations: df, number of observation‐fixed parameters (5).

^a^ Significant effect < 0.05.

Data processing is reported in line with Nolte et al. (2023) recommendations and adhered to Robergs et al. (2010) guidance, implemented using custom Python code. A two‐minute analysis window was set, ending 30 s before the end of the trial as participants were giving perceptual feedback impacting the VO_2_ measurement. An initial filter was applied to the data by excluding outliers ± 2 standard deviations (SD) from the mean of the analysis window. Missing data over a 50‐s period during the 2‐min window analysis resulted in the exclusion of the trial (3.5% of missing data for VO_2_). Similarly, if the connection between the heart rate sensor and the metabolic cart was reported as defective, participant data sets were excluded (20% of missing data for heart rate). The two trials of the same shoe were then averaged for each condition and participant, decreasing the noise of the measurement by 1/√*n* (*n* = 2) (Hopkins 2004; Buchheit 2014). If only one trial was valid for the participant, the valid measure was taken. Although the lactate check during the warm‐up ensured the athlete would be in a steady state for the running economy measurement, we checked that RER was < 1 for all participants (0.85 ± 0.06).

#### 2.5.2 Perceptual Metrics: Perceived Effort and Affect

To measure perceived effort while running, participants were asked to indicate ‘how hard, heavy and strenuous the physical task currently is’. Responses were provided according to the 15‐point rating of perceived exertion (RPE) scale (Borg 1998), with response options ranging from 6 ‘no exertion at all’ to 20 ‘maximal exertion’.

Core affect is defined as a neurophysiological state always available to consciousness but nonreflective (not needing to think about it to experience it). It was measured through the dimensions of valence (pleasure) and arousal (activation), which were assessed as per the circumplex model (Russell 1980). The Feeling Scale, developed by Hardy and Rejeski (1989), was used to measure affective valence. Participants were asked to rate how they felt during the running economy task using a single item rated on an 11‐point bipolar scale ranging from −5 (‘very bad’) to 5 (‘very good’), with anchors at −3 (‘bad’), −1 (‘fairly bad’), 0 (‘neutral’), 1 (‘fairly good’) and 3 (‘good’). Affective valence was explained as a feeling of pleasure/displeasure experienced while running. The arousal dimension was assessed using the Felt Arousal Scale (Svebak and Murgatroyd 1985). Participants were asked ‘How aroused are you?’ on a seven‐point scale ranging from 0 (‘low arousal’) to 6 (‘high arousal’). Arousal was explained as a feeling of activation that might be experienced in a variety of ways: high arousal as excitement or a feeling after drinking five cups of coffee and low arousal associated with feelings of relaxation or calmness. Instructions were provided before starting the protocol, with time to ask questions if needed regarding their understanding. Participants gave their ratings in the last 30 s of each running economy trial by pointing to the corresponding number on a printed scale.

### 2.6 Statistical Analysis

Data analysis was conducted using JASP (version 0.19.0; JASP; RRID:SCR_015823). The normality assumption was confirmed through the skewness and kurtosis test, Q‐Q plots and visual inspection. In order to analyse the effect of foam type on running economy and perceptual metrics at each running gradient, a general linear mixed model was used, as it allows for the inclusion of ordinal data. VO_2_, heart rate, RPE, affective valence and arousal were the dependent variables of interest analysed through a fixed effect of shoes, gradient and their interaction. A random effect grouping factor was added with participant number to account for interindividual differences only.

When a significant interaction effect was observed, paired *t*‐tests were used for post hoc comparisons. This was also used for exploratory analysis when interaction was close to being significant (*p* < 0.10). In the absence of interaction, the analysis focused on the main effect of shoe, as the effect of gradient on physiological parameters and RPE has already been extensively studied.

Because of the exploratory nature of integrating perceptual metrics in footwear testing, the main effect of gradient was also examined for valence and arousal. Wilcoxon signed‐rank tests were used for post hoc comparisons due to the ordinal nature of the data following a significant interaction or main effect of gradient.

Significance was set at 0.05 for all analyses. Cohen's d was calculated to determine effect sizes, with *d* = 0.2 to 0.5 considered small, *d* = 0.5 to 0.8 medium and *d* > 0.8 large (Cohen 1988).

## 3 Results

Oxygen consumption was lower when wearing the AFT‐f shoes (45.1 ± 6.3 mL.kg^−1^. min^−1^) compared to the TRADI‐f shoes (45.6 ± 6.6 mL.kg^−1^. min^−1^) regardless of gradient differences (*p* = 0.008; *d* = 0.47). This improvement equates to a 1.2% decrease in oxygen consumption when wearing the AFT‐f shoes. The interaction between gradient and shoes was not significant (*p* = 0.050). However, given that the *p*‐value was close to the significance threshold, we conducted exploratory post hoc analyses, which suggested a more pronounced improvement when wearing the AFT‐f shoes in the FLAT (+2.1%; *p* = 0.004) and UP (+1.0%; *p* = 0.029) conditions compared to DOWN (+0.2%; *p* = 0.876). Heart rate was lower when wearing the AFT‐f shoes (145 ± 17 bpm) compared to the TRADI‐f shoes (146 ± 17 bpm; *p* < 0.001; *d* = 1.19) regardless of gradient. Lower RPE was observed in the AFT‐f (10.8 ± 1.9) when compared to the TRADI‐f shoe (11.0 ± 2.0; *p* = 0.008; *d* = 0.43).

Affective valence was more positive when wearing the AFT‐f shoe (2.8 ± 1.3) compared to the TRADI‐f shoe (2.5 ± 1.4; *p* = 0.027; *d* = −0.39). No differences in arousal between shoes were found (*p* = 0.738; *d* = 0.06). No significant main effect of gradient was observed for affective valence (*p* = 0.676). Arousal differences were, however, identified between gradients (FLAT = 2.8 ± 0.9; UP = 2.8 ± 1.1; DOWN = 3.3 ± 1.3; *p* = 0.013), with post hoc tests indicating significantly higher arousal in the DOWN condition compared to both FLAT (*p* < 0.001) and UP (*p* = 0.003).

**FIGURE 4:**
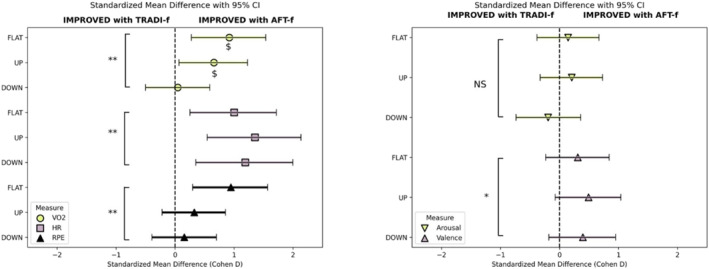
Standardised mean differences (Cohen's d) with 95% confidence intervals for VO_2_, heart rate (HR), RPE, arousal and affective valence across FLAT, UP and DOWN gradient conditions. Positive values (> 0, to the right of the reference line) indicate a favourable effect of the AFT‐f shoe, whereas negative values (< 0, to the left) indicate a favourable effect of the TRADI‐f shoe. Statistical significance across all gradients is indicated by *p* < 0.05 (*) and *p* < 0.01 (**). The ‘$’ symbol denotes a significant post hoc difference (*p* < 0.05) after an exploratory interaction effect.

All data are presented as mean ± standard deviation (SD) in Table 1 alongside statistical analyses. Standardised mean differences are visualised in Figure 4.

## 4 Discussion

The incorporation of AFT in trail running footwear has occurred despite uncertainty regarding the transferability of these technologies to footwear performance at different gradients. As a first investigation, this study aimed to explore whether the type of foam used in trail running footwear influences running economy and runners' perceived effort and affect in a laboratory setting. AFT foam was found to significantly improve running economy regardless of gradient, as demonstrated through a reduction in heart rate and a 1.2% decrease in oxygen consumption when compared to the TRADI foam. In addition to differences in running economy, lower perception of effort and higher pleasure were reported when running with the AFT foam. Although our results showed a tendency for larger improvements during level and uphill running, it is less clear how the foam impacted running economy in the downhill condition. Changes in arousal were identified between the different gradient conditions, supporting changes suggested in physiological measures. These insights provide an important development in the integration of AFT technologies for trail running footwear and performance.

Previous research in road running has reported up to 4% improvement in running economy with AFT (Hoogkamer et al. 2017), but this improvement is substantially lower when looking at the impact of foam only. For example, similar to the improvements that we observed, Worobets et al. (2014) reported a ∼1.0% improvement in running economy with a more compliant and resilient foam. However, such results have not been consistently observed, with Flores et al. (2018) finding that AFT foam did not improve running economy. These differences in findings might be explained by methodological differences such as speed, with evidence suggesting larger running economy gains from AFT at higher speeds (Rodrigo‐Carranza et al. 2022). Although the differences in gradient better reflect the demands of trail running, they make comparisons to previous work challenging. Future research considering the impact of AFT foam on running economy should consider the influence of speed and gradient.

The changes in running economy observed in this study were complemented by changes in athlete perceptions. Limited research has investigated the role of footwear on perceived effort and affective responses to running. Instead, existing research focuses on measures of participants' perceptions of the footwear itself and how these perceptions relate to running economy (Van Alsenoy et al. 2021). Given the practical involvement of these perceptions in running performance and training monitoring (Buchheit and Laursen 2013; Zenko and Ladwig 2021), it is surprising that they have not been commonly measured in footwear research. By integrating those metrics, we intend to assess more accurately the impact of AFT on trail running performance determinants, which goes beyond the measurement of running economy. Measuring affective valence also acknowledges what a successful performance is by looking at the experience of running in itself instead of the outcome only.

We observed that while running with AFT foam, runners reported both reduced perceived effort and more positive affective valence, without any change in arousal levels. Although the reasons for those simultaneous changes in perceptual metrics are unclear, we hypothesise that they could be related to either affective manipulation or change in effort intensity. In the first case, similar to studies using interventions such as music or caffeine, the higher pleasure while running with the AFT foam is likely to impact perceived effort (Terry et al. 2019; Astorino et al. 2012). In the second case, the increase in pleasure could be related to a lower effort intensity represented by a lower RPE procured by the shoe (Ekkekakis et al. 2020).

Practical applications can be drawn from the results of this study. In recent international trail running races, the gap between first and second place has often been around 1 minute (e.g., 1 min 11s at MCC 2023; 46s at Marathon du Mont‐Blanc Results 2024; 1 min 38s at Marathon du Mont‐Blanc 90k 2023). Although improvements in running economy of similar magnitude (∼1%) have been associated with ∼0.8% reduction in racing time in 3000 m events (Hoogkamer et al. 2016), this has not been confirmed in trail running contexts. Further investigations are needed to determine whether such improvements translate into meaningful performance gains in trail environments. From a footwear design perspective, a 1% improvement in running economy has an effect equivalent to reducing shoe mass by 100 g (Rodrigo‐Carranza et al. 2020), which highlights the potential impact of AFT foams on performance outcomes.

**FIGURE 5:**
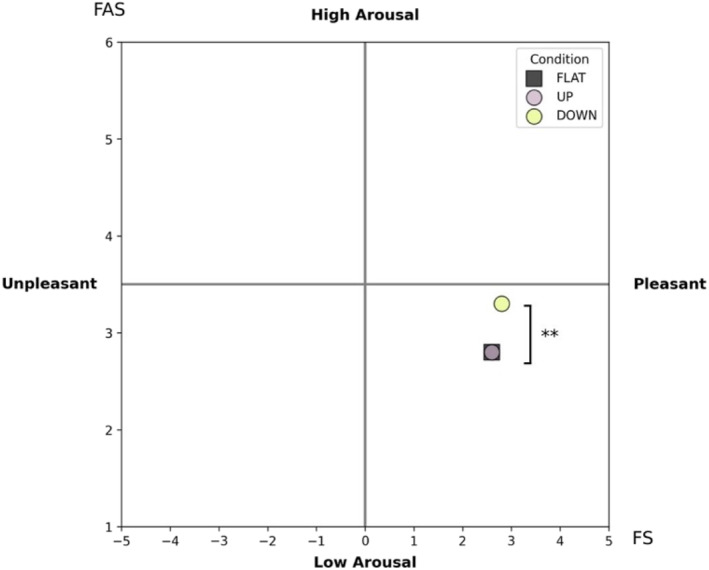
Affective valence (FS) and arousal (FAS) plotted within the circumplex model of affect for the FLAT, UP and DOWN gradient conditions. Each point represents the mean affective state for each condition, independent of shoe condition. A significant difference in arousal between the DOWN and UP/FLAT conditions is indicated by ** (*p* < 0.01).

Although the AFT foam appeared to improve running economy, it is unclear how this improvement was affected by the three gradients used in this study. Exploratory analyses were conducted to better understand the phenomenon despite the absence of an interaction effect between gradient and shoe condition (*p* = 0.050). Our data provide some evidence for greater improvements in running economy when running on level and uphill conditions but not downhill (Figure 4). We observed large variability in physiological indices and high arousal levels during downhill running (Figure 5). The high arousal levels may be related to the treadmill setting, interfering with runners' ability to judge the length of the treadmill belt. Participants reported the downhill condition to be unnatural and making them nervous. High arousal has been shown to impact breathing metabolic rate (Migliaccio et al. 2023) and, as such, we believe the high arousal levels may have interfered with gaining representative physiological loads for the downhill condition. Further research is required to support this observation and account for our observations during downhill running.

Although changes in arousal induced by the protocol setting may interfere with results, other studies have reported changes in running economy when running across different elevation gradients. Whiting et al. (2021) reported an improvement in both levelled and uphill conditions, although the benefits were notably smaller during uphill running (∼1% difference) when testing footwear including both plate and foam elements. In the present study, only the foam component was manipulated. Foam properties likely contributed to improved running economy, especially under conditions where the foam can deform sufficiently to release stored elastic energy (Burns and Joubert 2024; Joubert et al. 2023).

In contrast, plate technologies restrict metatarsophalangeal joint flexion. Although this may be beneficial on level running, Jaboulay and Giandolini (2025) demonstrated that such stiffness can become detrimental during uphill running, where natural foot mechanics require more dorsiflexion. Although our study did not include the manipulation of plate properties, the tendency for improved running economy observed uphill may stem from foam properties alone, potentially due to their ability to deform and return energy without restricting foot motion.

### 4.1 Limitations

The findings of this paper should be interpreted in light of several limitations. Firstly, due to time constraints, athlete performance levels and shoe size availability, the final sample was limited to 14 participants, despite having recruited 18. A sample size estimation based on data from Hoogkamer et al. (2017) suggested that 14 participants would provide 80% power to detect differences in the main shoe effect (*f* = 0.38) using a repeated‐measures, within‐subjects design. Although our sample met this estimate, the calculation relied on several assumptions, focused on a single metric (the primary aim) and did not account for other aims, including perceptual metrics. As such, future studies, particularly those seeking to understand perceptual responses, should aim for larger sample sizes. Additionally, restricting recruitment to a single shoe size limited participants to males, which may affect the generalisability of the results to female populations.

An apparent limitation of this laboratory study was the measurement of responses during the downhill condition. Familiarisation sessions would likely be required to improve the reliability of responses in this condition.

Finally, limitations also concern the perceptual metrics. Participants were not blinded to shoe design, and the visible colour difference between the two shoes may have influenced their responses. Furthermore, wearing a respiratory mask may have affected participants' affective state. Although the perceptual scales used were validated in English, most participants were not native English speakers, and some reported difficulty understanding the meaning of arousal, which could explain the inability to detect changes between shoe conditions.

Future research investigating the physiological and perceptual effects of AFT across varying gradients would benefit from ecologically valid protocols conducted in real‐world environments, better reflecting the technical demands of trail running.

## 5 Conclusion

The findings of this study suggest that AFT foam in trail running shoes can improve running economy and perceived effort and increase pleasure while running at different gradients. A tendency for greater improvement in running economy in levelled and uphill running was identified, although the results from the downhill condition were unclear. The changes in running economy and perception appear to be meaningful and provide scientific evidence to better inform the development of specific trail running footwear, although they must be taken under the context of a well‐trained athlete population. Finally, we encourage future research to integrate perceptual measurements related to effort to better understand the AFT phenomenon, particularly in trail running. Ecologically valid studies conducted in real‐world environments are also needed to confirm these findings and better reflect the complex demands of trail running.

## Ethics Statement

This study was approved by the Human Research Ethics Committee from the University of Canberra (Grant No. 202413976). All procedures were conducted in accordance with the Declaration of Helsinki.

## Consent

All participants provided written informed consent before taking part in this study.

## Conflicts of Interest

The authors declare no conflicts of interest.

## References

[ejsc70059-bib-0001] Astorino, T. A. , T. Cottrell , A. T. Lozano , K. Aburto‐Pratt , and J. Duhon . 2012. “Effect of Caffeine on RPE and Perceptions of Pain, Arousal, and Pleasure/Displeasure During a Cycling Time Trial in Endurance Trained and Active Men.” Physiology & Behavior 106, no. 2: 211‐217. 10.1016/j.physbeh.2012.02.006. 22349482

[ejsc70059-bib-0002] Balducci, P. , M. Clémençon , R. Trama , Y. Blache , and C. Hautier . 2017. “Performance Factors in a Mountain Ultramarathon.” International Journal of Sports Medicine 38, no. 11: 819–826. 10.1055/s-0043-112342. 28799161

[ejsc70059-bib-0003] Barnes, K. R. , and A. E. Kilding . 2015. “Running Economy: Measurement, Norms, and Determining Factors.” Sports Medicine—Open 1, no. 1: 8. 10.1186/s40798-015-0007-y. 27747844 PMC4555089

[ejsc70059-bib-0004] Barrons, Z. B. , V. Rodrigo‐Carranza , M. Bertschy , and W. Hoogkamer . 2024. “The Fallacy of Single Trials: The Need for Multiple Trials in Assessing Running Economy Responses in Advanced Footwear Technology.” Sports Medicine 54, no. 6: 1357–1360. 10.1007/s40279-023-01991-1. 38407747

[ejsc70059-bib-0005] Beijst, C. , G. Schep , E. Van Breda , P. F. F. Wijn , and C. Van Pul . 2012. “Accuracy and Precision of CPET Equipment: A Comparison of Breath‐by‐Breath and Mixing Chamber Systems.” Journal of Medical Engineering & Technology 37, no. 1: 35–42. 10.3109/03091902.2012.733057. 23110656

[ejsc70059-bib-0006] Bishop, C. , J. D. Buckley , A. E. Esterman , and J. B. Arnold . 2020. “The Running Shoe Comfort Assessment Tool (RUN‐CAT): Development and Evaluation of a New Multi‐Item Assessment Tool for Evaluating the Comfort of Running Footwear.” Journal of Sports Sciences 38, no. 18: 2100–2107. 10.1080/02640414.2020.1773613. 32508250

[ejsc70059-bib-0007] Blanchfield, A. , J. Hardy , and S. Marcora . 2014. “Non‐Conscious Visual Cues Related to Affect and Action Alter Perception of Effort and Endurance Performance.” Frontiers in Human Neuroscience 8: 967. 10.3389/fnhum.2014.00967. 25566014 PMC4263011

[ejsc70059-bib-0008] Borg, G. 1998. Borg’s Perceived Exertion and Pain Scales. Human Kinetics.

[ejsc70059-bib-0009] Buchheit, M. 2014. “Monitoring Training Status With HR Measures: Do all Roads Lead to Rome.” Frontiers in Physiology 5: 73. 10.3389/fphys.2014.00073. 24578692 PMC3936188

[ejsc70059-bib-0010] Buchheit, M. , and P. B. Laursen . 2013. “High‐Intensity Interval Training, Solutions to the Programming Puzzle.” Sports Medicine 43, no. 5: 313–338. 10.1007/s40279-013-0029-x. 23539308

[ejsc70059-bib-0011] Burns, G. T. , and D. P. Joubert . 2024. “Running Shoes of the Postmodern Footwear Era: A Narrative Overview of Advanced Footwear Technology.” International Journal of Sports Physiology and Performance 19, no. 10: 975‐986–986. 10.1123/ijspp.2023-0446. 39117307

[ejsc70059-bib-0012] Carmo, E. C. D. , R. Barroso , A. Renfree , N. R. Da Silva , S. Gil , and V. Tricoli . 2020. “Affective Feelings and Perceived Exertion During a 10‐km Time Trial and head‐to‐head Running Race.” International Journal of Sports Physiology and Performance 15, no. 6: 903–906. 10.1123/ijspp.2019-0586. 32050163

[ejsc70059-bib-0013] Chollet, M. , S. Michelet , N. Horvais , S. Pavailler , and M. Giandolini . 2022. “Individual Physiological Responses to Changes in Shoe Bending Stiffness: A Cluster Analysis Study on 96 Runners.” European Journal of Applied Physiology 123, no. 1: 169–177. 10.1007/s00421-022-05060-9. 36229743

[ejsc70059-bib-0014] Coates, A. M. , J. A. Berard , T. J. King , and J. F. Burr . 2021. “Physiological Determinants of Ultramarathon Trail‐Running Performance.” International Journal of Sports Physiology and Performance 16, no. 10: 1454–1461. 10.1123/ijspp.2020-0766. 33691287

[ejsc70059-bib-0015] Cohen, J. 1988. Statistical Power Analysis for the Behavioral Sciences. 2^e^ éd. Lawrence Erlbaum Associates.

[ejsc70059-bib-0016] Colosio, A. L. , K. Caen , J. G. Bourgois , J. Boone , and S. Pogliaghi . 2020. “Bioenergetics of the VO2 Slow Component Between Exercise Intensity Domains.” Pfluegers Archiv European Journal of Physiology 472, no. 10: 1447–1456. 10.1007/s00424-020-02437-7. 32666276 PMC7476983

[ejsc70059-bib-0017] Day, E. , and M. Hahn . 2019. “Optimal Footwear Longitudinal Bending Stiffness to Improve Running Economy Is Speed Dependent.” Footwear Science 12, no. 1: 3–13. 10.1080/19424280.2019.1696897.

[ejsc70059-bib-0018] Ehrström, S. , M. P. Tartaruga , C. S. Easthope , J. Brisswalter , J. Morin , and F. Vercruyssen . 2017. “Short Trail Running Race.” Medicine & Science in Sports & Exercise 50, no. 3: 580–588. 10.1249/mss.0000000000001467. 29077639

[ejsc70059-bib-0019] Ekkekakis, P. , M. E. Hartman , and M. A. Ladwig . 2020. “Affective Responses to Exercise.” In Handbook of Sport Psychology. 4th ed., 231–253. 10.1002/9781119568124.ch12.

[ejsc70059-bib-0020] Exercise & Sports Science Australia . 2018. Adult Pre‐Exercise Screening System (APSS) Version 2. https://www.essa.org.au/Web/Web/Resources/Tools‐and‐templates/Adult‐Pre‐Exercise‐Screening‐System‐User‐Guide.aspx.

[ejsc70059-bib-0021] Flores, N. , N. Delattre , E. Berton , and G. Rao . 2018. “Does an Increase in Energy Return and/or Longitudinal Bending Stiffness Shoe Features Reduce the Energetic Cost of Running.” European Journal of Applied Physiology 119, no. 2: 429–439. 10.1007/s00421-018-4038-1. 30470873

[ejsc70059-bib-0022] Frederick, E. C. 2022. “Let’s Just Call It Advanced Footwear Technology (AFT).” Footwear Science 14, no. 3: 131. 10.1080/19424280.2022.2127526.

[ejsc70059-bib-0023] Hardy, C. J. , and W. J. Rejeski . 1989. “Not What, But How One Feels: The Measurement of Affect During Exercise.” Journal of Sport & Exercise Psychology 11, no. 3: 304–317. 10.1123/jsep.11.3.304.

[ejsc70059-bib-0024] Hébert‐Losier, K. , H. Knighton , S. J. Finlayson , B. Dubois , J. Esculier , and C. M. Beaven . 2023. “Biomechanics and Subjective Measures of Recreational Male Runners in Three Shoes Running Outdoors: A Randomised Crossover Study.” Footwear Science 16, no. 1: 13‐23. 10.1080/19424280.2023.2283460.

[ejsc70059-bib-0025] Hébert‐Losier, K. , and M. Pamment . 2022. “Advancements in Running Shoe Technology and Their Effects on Running Economy and Performance – A Current Concepts Overview.” Sports Biomechanics 22, no. 3: 335–350. 10.1080/14763141.2022.2110512. 35993160

[ejsc70059-bib-0026] Hoogkamer, W. , S. Kipp , J. H. Frank , E. M. Farina , G. Luo , and R. Kram . 2017. “A Comparison of the Energetic Cost of Running in Marathon Racing Shoes.” Sports Medicine 48, no. 4: 1009–1019. 10.1007/s40279-017-0811-2. PMC5856879 29143929

[ejsc70059-bib-0027] Hoogkamer, W. , S. Kipp , B. A. Spiering , and R. Kram . 2016. “Altered Running Economy Directly Translates to Altered Distance‐Running Performance.” Medicine & Science in Sports & Exercise 48, no. 11: 2175–2180. 10.1249/mss.0000000000001012. 27327023

[ejsc70059-bib-0028] Hopkins, W. G. 2004. “How to Interpret Changes in an Athletic Performance Test.” Sportscience 8: 1–7. https://www.sportsci.org/jour/04/wghtests.htm.

[ejsc70059-bib-0029] Hunter, I. , A. McLeod , D. Valentine , T. Low , J. Ward , and R. Hager . 2019. “Running Economy, Mechanics, and Marathon Racing Shoes.” Journal of Sports Sciences 37, no. 20: 2367–2373. 10.1080/02640414.2019.1633837. 31223054

[ejsc70059-bib-0030] Jaboulay, C. , and M. Giandolini . 2025. “Effect of Increased Bending Stiffness on Running Economy and Joint Biomechanics in Uphill Running and Running on Unstable Terrain: Is There Any Evidence for Embedding Carbon Plate in Trail Running Footwear.” Footwear Science 1–9, no. 1: 19–27. 10.1080/19424280.2024.2448653.

[ejsc70059-bib-0031] Joubert, D. P. , T. A. Dominy , and G. T. Burns . 2023. “Effects of Highly Cushioned and Resilient Racing Shoes on Running Economy at Slower Running Speeds.” International Journal of Sports Physiology and Performance 18, no. 2: 164–170. 10.1123/ijspp.2022-0227. 36626911

[ejsc70059-bib-0032] Joyner, M. J. , and E. F. Coyle . 2007. “Endurance Exercise Performance: The Physiology of Champions.” Journal Of Physiology 586, no. 1: 35–44. 10.1113/jphysiol.2007.143834. 17901124 PMC2375555

[ejsc70059-bib-0033] Langley, J. O. , and B. Langley . 2023. “The Effect of Advanced Footwear Technology on Elite Male Marathon Race Speed.” European Journal of Applied Physiology 124, no. 4: 1143–1149. 10.1007/s00421-023-05341-x. 37922023

[ejsc70059-bib-0034] Marathon du Mont‐Blanc Results . 2024. Official Results ‐ Marathon Du Mont‐Blanc 2024. https://www.marathonmontblanc.fr/resultats.

[ejsc70059-bib-0035] Marcora, S. M. , W. Staiano , and V. Manning . 2009. “Mental Fatigue Impairs Physical Performance in Humans.” Journal of Applied Physiology 106, no. 3: 857–864. 10.1152/japplphysiol.91324.2008. 19131473

[ejsc70059-bib-0036] Matthias, E. C. , H. A. Banwell , and J. B. Arnold . 2021. “Methods for Assessing Footwear Comfort: A Systematic Review.” Footwear Science 13, no. 3: 255–274. 10.1080/19424280.2021.1961879.

[ejsc70059-bib-0037] McKay, A. K. , T. Stellingwerff , E. S. Smith , et al. 2021. “Defining Training and Performance Caliber: A Participant Classification Framework.” International Journal of Sports Physiology and Performance 17, no. 2: 317–331. 10.1123/ijspp.2021-0451. 34965513

[ejsc70059-bib-0038] McLaughlin, J. E. , E. T. Howley , D. R. Bassett , D. L. Thompson , and E. C. Fitzhugh . 2010. “Test of the Classic Model for Predicting Endurance Running Performance.” Medicine & Science in Sports & Exercise 42, no. 5: 991–997. 10.1249/mss.0b013e3181c0669d. 19997010

[ejsc70059-bib-0039] Migliaccio, G. M. , L. Russo , M. Maric , and J. Padulo . 2023. “Sports Performance and Breathing Rate: What Is the Connection? A Narrative Review on Breathing Strategies.” Sports 11, no. 5: 103. 10.3390/sports11050103. 37234059 PMC10224217

[ejsc70059-bib-0040] Millet, G. Y. 2011. “Can Neuromuscular Fatigue Explain Running Strategies and Performance in Ultra‐Marathons.” Sports Medicine 41, no. 6: 489–506. 10.2165/11588760-000000000-00000. 21615190

[ejsc70059-bib-0041] Mills, K. , P. Blanch , and B. Vicenzino . 2010. “Identifying Clinically Meaningful Tools for Measuring Comfort Perception of Footwear.” Medicine & Science in Sports & Exercise 42, no. 10: 1966–1971. 10.1249/mss.0b013e3181dbacc8. 20216463

[ejsc70059-bib-0042] Nolte, S. , R. Rein , and O. J. Quittmann . 2023. “Data Processing Strategies to Determine Maximum Oxygen Uptake: A Systematic Scoping Review and Experimental Comparison With Guidelines for Reporting.” Sports Medicine 53, no. 12: 2463–2475. 10.1007/s40279-023-01903-3. 37603201 PMC10687136

[ejsc70059-bib-0043] Pageaux, B. 2014. “The Psychobiological Model of Endurance Performance: An Effort‐Based Decision‐Making Theory to Explain Self‐Paced Endurance Performance.” Sports Medicine 44, no. 9: 1319–1320. 10.1007/s40279-014-0198-2. 24809249

[ejsc70059-bib-0044] Perez‐Suarez, I. , M. Martin‐Rincon , J. J. Gonzalez‐Henriquez , et al. 2018. “Accuracy and Precision of the COSMED K5 Portable Analyser.” Frontiers in Physiology 9: 1764. 10.3389/fphys.2018.01764. 30622475 PMC6308190

[ejsc70059-bib-0045] Péronnet, F. , and D. Massicotte . 1991. “Table of Nonprotein Respiratory Quotient: An Update.” Canadian Journal of Sport Sciences 16, no. 1: 23–29. 1645211

[ejsc70059-bib-0046] Renfree, A. , J. West , M. Corbett , C. Rhoden , and A. St Clair Gibson . 2012. “Complex Interplay Between Determinants of Pacing and Performance During 20‐km Cycle Time Trials.” International Journal of Sports Physiology and Performance 7, no. 2: 121–129. 10.1123/ijspp.7.2.121. 22173069

[ejsc70059-bib-0047] Robergs, R. A. , D. Dwyer , and T. Astorino . 2010. “Recommendations for Improved Data Processing From Expired Gas Analysis Indirect Calorimetry.” Sports Medicine 40, no. 2: 95–111. 10.2165/11319670-000000000-00000. 20092364

[ejsc70059-bib-0048] Rodrigo‐Carranza, V. , F. González‐Mohíno , J. Santos‐Concejero , and J. M. González‐Ravé . 2020. “Influence of Shoe Mass on Performance and Running Economy in Trained Runners.” Frontiers in Physiology 11: 573660. 10.3389/fphys.2020.573660. 33071828 PMC7538857

[ejsc70059-bib-0049] Rodrigo‐Carranza, V. , F. González‐Mohíno , J. Santos‐Concejero , and J. M. González‐Ravé . 2022. “Impact of Advanced Footwear Technology on Elite Men’s in the Evolution of Road Race Performance.” Journal of Sports Sciences 40, no. 23: 2661–2668. 10.1080/02640414.2023.2183103. 36814065

[ejsc70059-bib-0050] Russell, J. A. 1980. “A Circumplex Model of Affect.” Journal of Personality and Social Psychology 39, no. 6: 1161–1178. 10.1037/h0077714.

[ejsc70059-bib-0051] Saunders, P. U. , D. B. Pyne , R. D. Telford , and J. A. Hawley . 2004. “Factors Affecting Running Economy in Trained Distance Runners.” Sports Medicine 34, no. 7: 465–485. 10.2165/00007256-200434070-00005. 15233599

[ejsc70059-bib-0052] Scheer, V. , T. I. Janssen , S. Vieluf , and H. Heitkamp . 2018. “Predicting Trail‐Running Performance With Laboratory Exercise Tests and Field‐Based Results.” International Journal of Sports Physiology and Performance 14, no. 1: 130–133. 10.1123/ijspp.2018-0390. 29952678

[ejsc70059-bib-0053] Svebak, S. , and S. Murgatroyd . 1985. “Metamotivational Dominance: A Multimethod Validation of Reversal Theory Constructs.” Journal of Personality and Social Psychology 48, no. 1: 107–116. 10.1037/0022-3514.48.1.107.

[ejsc70059-bib-0054] Terry, P. C. , C. I. Karageorghis , M. L. Curran , O. V. Martin , and R. L. Parsons‐Smith . 2019. “Effects of Music in Exercise and Sport: A Meta‐Analytic Review.” Psychological Bulletin 146, no. 2: 91–117. 10.1037/bul0000216. 31804098

[ejsc70059-bib-0055] Tucker, R. 2009. “The Anticipatory Regulation of Performance : The Physiological Basis for Pacing Strategies and the Development of a Perception‐Based Model for Exercise Performance.” British Journal of Sports Medicine 43, no. 6: 392–400. 10.1136/bjsm.2008.050799. 19224911

[ejsc70059-bib-0056] Van Alsenoy, K. , M. L. Van Der Linden , O. Girard , and D. Santos . 2021. “Increased Footwear Comfort Is Associated With Improved Running Economy – A Systematic Review and Meta‐Analysis.” European Journal of Sport Science 23, no. 1: 121–133. 10.1080/17461391.2021.1998642. 34726119

[ejsc70059-bib-0057] Ward, S. A. 2018. “Open‐Circuit Respirometry: Real‐Time, Laboratory‐Based Systems.” European Journal of Applied Physiology 118, no. 5: 875–898. 10.1007/s00421-018-3860-9. 29728765

[ejsc70059-bib-0058] Whiting, C. S. , W. Hoogkamer , and R. Kram . 2021. “Metabolic Cost of Level, Uphill, and Downhill Running in Highly Cushioned Shoes With Carbon‐Fiber Plates.” Journal of Sport and Health Science 11, no. 3: 303–308. 10.1016/j.jshs.2021.10.004. 34740871 PMC9189710

[ejsc70059-bib-0059] Worobets, J. , J. W. Wannop , E. Tomaras , and D. Stefanyshyn . 2014. “Softer and More Resilient Running Shoe Cushioning Properties Enhance Running Economy.” Footwear Science 6, no. 3: 147–153. 10.1080/19424280.2014.918184.

[ejsc70059-bib-0060] Zenko, Z. , and M. Ladwig . 2021. “Affective Responses to Exercise: Measurement Considerations for Practicing Professionals.” In Dans Society for Transparency, Openness, and Replication in Kinesiology Ebooks, 271–293. 10.51224/b1012.

